# Optimum Use of Acute Treatments for Hereditary Angioedema: Evidence-Based Expert Consensus

**DOI:** 10.3389/fmed.2017.00245

**Published:** 2018-03-12

**Authors:** Hilary Longhurst

**Affiliations:** ^1^Honorary Consultant Immunologist, Department of Clinical Biochemistry and Immunology, Addenbrooke’s Hospital, Cambridge University Hospitals NHS Foundation Trust, Cambridge, United Kingdom

**Keywords:** angioedemas, hereditary, C1 inhibitor, acute therapy, icatibant, ecallantide, recombinant C1 inhibitor

## Abstract

Acute treatment of hereditary angioedema due to C1 inhibitor deficiency has become available in the last 10 years and has greatly improved patients’ quality of life. Two plasma-derived C1 inhibitors (Berinert and Cinryze), a recombinant C1 inhibitor (Ruconest/Conestat alpha), a kallikrein inhibitor (Ecallantide), and a bradykinin B2 receptor inhibitor (Icatibant) are all effective. Durably good response is maintained over repeated treatments and several years. All currently available prophylactic agents are associated with breakthrough attacks, therefore an acute treatment plan is essential for every patient. Experience has shown that higher doses of C1 inhibitor than previously recommended may be desirable, although only recombinant C1 inhibitor has been subject to full dose–response evaluation. Treatment of early symptoms of an attack, with any licensed therapy, results in milder symptoms, more rapid resolution and shorter duration of attack, compared with later treatment. All therapies have been shown to be well-tolerated, with low risk of serious adverse events. Plasma-derived C1 inhibitors have a reassuring safety record regarding lack of transmission of virus or other infection. Thrombosis has been reported in association with plasma-derived C1 inhibitor in some case series. Ruconest was associated with anaphylaxis in a single rabbit-allergic volunteer, but no further anaphylaxis has been reported in those not allergic to rabbits despite, in a few cases, prior IgE sensitization to rabbit or milk protein. Icatibant is associated with high incidence of local reactions but not with systemic effects. Ecallantide may cause anaphylactoid reactions and is given under supervision. For children and pregnant women, plasma-derived C1 inhibitor has the best evidence of safety and currently remains first-line treatment.

## Introduction

Hereditary angioedema, due to C1 inhibitor deficiency, is a disabling and sometimes fatal disorder. Unpredictable swelling affects cutaneous or mucosal sites, causing pain, disfigurement, and disability, which lasts several days (1, 2). Acute treatment reduces severity and duration of attack for most patients, whose quality of life has improved since such treatments became available (3–5).

Double-blind studies of acute therapies have been challenging: factors such as natural variability of angioedema, spontaneous resolution after 1–5 days, high-placebo response, small patient numbers, subjective endpoints, and differences in use of symptom-reducing treatments contributing to the difficulty. Moreover, double-blind studies were carried out in hospital, with treatment given relatively late, at 4–7 h after onset. The requirement for participants to travel to the study center, rather than self-administering or attending their local hospital, may well also have resulted in additional bias.

Angioedema of HAE may be preceded by prodromal symptoms of erythema marginatum, fatigue, tingling, or irritability in around 25% of attacks (6). These early indicators of subclinical angioedema are superceded within hours by low-level non-specific symptoms, for example, abdominal discomfort in the case of abdominal attacks or tightness in the case of cutaneous or laryngeal attacks (7, 8). Although many patients correctly interpret these symptoms as early angioedema, there is potential for misattribution both of true angioedema-related symptoms to another cause and for other causes of the symptoms to be falsely attributed to HAE. In the case of an HAE attack, symptoms typically increase exponentially in severity after several hours, remaining severe for up to 24 h before resolving over the subsequent 1–2 days if untreated (9).

At the time of the studies, emphasis was largely on relief of severe symptoms. Since this time, expert opinion has shifted to focus on the prevention of pain and disability that arises from severe symptoms, in order to allow uninterrupted work, education or other activities, and to optimize quality of life (10–14). For this reason, the time to complete or almost complete resolution has gained in importance compared with the time to initial improvement (11). This focused review reports the discussions and recommendations, relating to treatment of acute angioedema attacks in HAE, made at a meeting of the HAWK group of experts and stakeholders, which took place in September 2016 in Gargnano, Italy.

## Methods

We considered evidence relating to essential considerations of efficacy and safety of available therapies. For agents considered efficacious and safe, we attempted to go further, to consider other factors impacting on effectiveness, patient experience, and outcomes. We therefore considered additional evidence on timing of treatment, dose of C1 inhibitor, and potential need for retreatment, as well as evidence relating to special clinical situations or vulnerable patients such as children, pregnant, or breast-feeding women. Organizational factors, including the importance of access to self-administration of acute treatment, are discussed in elsewhere in this issue [(15)—this issue].

I conducted a systematic review using the search terms “hereditary angioedema,” “hereditary angioneurotic (o)edema,” “hereditary angioedema,” “C1 inhibitor deficiency,” “C1 esterase inhibitor deficiency,” and “therapy” in September 2016. Publications thus identified were used to inform discussion and statements concerning best acute treatment strategy were agreed. Additional evidence, published during the preparation of this review, is also referenced.

## Results

### Efficacy

Double-blind studies treating established moderate or severe attacks demonstrate that duration and severity can be reduced. Initial improvement may be delayed several hours, and full relief hours or days, after treatment. All studies reported high placebo response, most likely due to subjective endpoints, spontaneous resolution of some attacks and in some trials, differences in use of non-specific measures such as rehydration and analgesia (16–26). Nevertheless, most studies showed superiority over placebo in reducing time to improvement. Active treatment was also associated with a greater proportion of attacks with definitive response at 4 h.

Double-blind and observational studies have demonstrated the efficacy of plasma-derived and recombinant C1 inhibitors, a kallikrein inhibitor, and a bradykinin B2 receptor inhibitor in treating acute HAE-related angioedema attacks (16–26) (Table 1).

**Table 1:** Acute treatments for hereditary angioedema.

Name	Mode of action	Recommended dose	Route of administration	Self/home administration?	Comments
Berinert	Plasma-derived C1 inhibitor	20 U/kg	Intravenous	Yes	C1 inhibitor is first choice in pregnancy. Extensive experience in children
Cinryze	Plasma-derived C1 inhibitor	1,000 U with additional 1,000 U given at physician’s discretion	Intravenous	Yes	C1 inhibitor is first choice in pregnancy
Ruconest (Conestat alpha)	Recombinant C1 inhibitor	50 U/kg or 4,200 U if >84 kg	Subcutaneous	Yes	Contraindicated in clinical rabbit allergy
Icatibant (Firazyr)	Bradykinin B2 receptor antagonist	30 mg	Subcutaneous	Yes	Avoid in acute thrombosis. Local reactions common
Ecallantide (Kalbitor)	Kallikrein antagonist	30 mg	Subcutaneous	No (administered by home-visit nurse)	Risk of anaphylactoid event

#### Double-Blind Studies

Studies of plasma-derived C1 inhibitor include a study of a vapor-heated, pressure-treated C1 inhibitor (immuno, no longer available). Patients with moderate or angioedema at any site, treated within 5 h of onset showed initial improvement after a mean (SD) of 55 (±16) min compared with 563 (±72) min for those treated with albumin placebo. Full resolution occurred after a mean (SD) 23.98 (14.81) h compared with 34.58 (13.56) h for placebo. 95% of those treated with C1 inhibitor had responded within 4 h, compared with 12% of those given placebo. 28 of 49 placebo-treated attacks required C1 inhibitor within 4 h, compared with none of 55 C1 inhibitor-treated attacks (16, 17).

An unpublished study of a similar C1 inhibitor product measuring improvement 1 h after double-blind administration of C1 inhibitor or albumin failed to show significant benefit of C1 inhibitor, although an open-label study of the same C1 inhibitor appeared to show benefit (Baxter, unpublished data on file). This apparent treatment failure is likely to represent inappropriate study design, which did not take into account the relatively slow onset of symptomatic benefit, particularly when severe attacks are treated late, as is likely to have been the case here.

More recent studies of nanofiltered plasma-derived C1 inhibitor have confirmed benefit in acute treatment. Cinryze 1,000 U, administered within 4 h of onset of symptoms at any non-laryngeal site, with an additional 1,000 U given at 1 h at the physician’s discretion if improvement was insufficient, resulted in initial improvement at 4 h in 21 of 35 subjects (60%) compared with 14 of 33 subjects (40%) treated with one or two doses of placebo (*p* = 0.06). Median time to onset of relief, defined as the first of three consecutive reports of improvement, was 2 h with Cinryze, compared with over 4 h for placebo (*p* = 0.02). 23 of 35 subjects receiving C1 inhibitor and 28 of 33 of subjects receiving placebo were given a second 1,000 U dose of Cinryze or further placebo treatment, respectively. Complete resolution occurred after a median of 12.3 and 25 h in Cinryze and placebo groups, respectively (*p* = 0.04), despite rescue Cinryze (1,000 U) administered to subjects who had not experienced onset of relief at 4 h (21).

Likewise, Berinert, a plasma-derived C1 inhibitor, administered at 20 U/kg within 5 h of onset, to 43 subjects with moderate or severe abdominal pain or facial swelling, achieved onset of improvement at a median (range) 0.5 (0.07–24) h, compared with 1.5 (0.17–24) h for 42 subjects treated with placebo (*p* = 0.0025). 37 of 43 (86%) of subjects treated with Berinert noted improvement at 4 h, compared with 25 of 42 (59.5%) treated with placebo. Complete relief was noted at a median (range) 4.92 (0.47–1,486) h, compared with 7.79 (0.33–1,486) h in the placebo group, despite subjects who did not report relief at 4 h being crossed over to receive placebo or C1 inhibitor for a total of 20 U/kg in the 4-h period. 39 subjects who received a dose of 10 U/kg showed a non-significant trend favoring Berinert (18).

Units of recombinant C1 inhibitor (Conestat alpha; Ruconest) have equal potency with plasma-derived C1 inhibitor, but a shorter half-life (27). Unlike the plasma-derived C1 inhibitors, dose–response studies have been carried out for recombinant C1 inhibitor, showing optimum response at 50–100 U/kg (21, 25). Recombinant C1 inhibitor 50–100 U/kg showed efficacy when given within 8 h of onset of an attack at any site. Initial improvement at 4 h was seen in 90–100% of those treated with 100 and 50 U/kg, respectively, compared with 41% treated with saline placebo. Median time [95% confidence interval (CI)] to onset of improvement was 1.1 (1–2.03) h for 26 patients treated with 100 U/kg, 2.03 (1.2–2.25) h for 15 treated with 50 U/kg, and 8.25 (4.08–7.66) h for 29 treated with placebo (*p* < 0.01/*p* = 0.013 for 100/50 U, respectively, compared with placebo) (21). A further study showed median (95% CI) initial response in 1.5 (1.0–2.5) h compared with 2.5 (1.56 to >4) h for 44 patients treated with 50 U/kg and 33 who received placebo, respectively (*p* = 0.031). In this study, almost full resolution was achieved in 4.0 (2.95–4.5) h for recombinant C1 inhibitor and 6.03 (4 to >48) h for placebo (*p* = 0.05) (25).

Icatibant, a bradykinin B2 receptor antagonist, showed efficacy for 106 patients over three trials (FAST 1–3), within 6 h of onset of moderate or severe attacks at any non-laryngeal site. The dose was 30 mg, given subcutaneously. Initial improvement occurred at 4 h in 67% (CI 46–84%) of 27 and 43 subjects (FAST 1 and 3, respectively) compared with 46% (CI 28–66%) treated with placebo (*p* = 0.18). 80% (CI 63–92%) of 36 subjects treated with icatibant improved within 4 h compared with 31% (CI 116–48%) of 38 subjects treated with tranexamic acid comparator (*p* < 0.01; FAST 2). Median (IQR) time to initial improvement was 1.5 (0.5–2) 2 (1.5–3) and 0.8 h with icatibant in FAST 1–3, respectively, compared with 19.8 (2.5—not evaluable) and 19.8 (6.1–26.3) h for placebo (*p* < 0.001), and 0.8 (0.4–1.4) h for icatibant compared with 7.9 (1.1—not evaluable) h for tranexamic acid (*p* < 0.001).

Likewise, subjects treated with icatibant had minimal symptoms after a median (IQR) 8.5 (2.5–31.15) h compared with 19.4 (10.2–55.7) h for placebo (*p* = 0.08) and 10.0 (2.8–23.2) h compared with 51.0 (12–79.5) h for tranexamic acid (*p* < 0.001). The use of rescue medication and supportive treatment was much higher in the placebo/tranexamic acid arms and this may have contributed to the lack of significance in the primary endpoint of significant (>30% improvement) relief for the FAST 1 trial (19, 26).

Ecallantide, a kallikrein antagonist, given within 8 h of onset of moderate or severe abdominal pain or facial swelling, resulted in initial improvement at 4 h in 69% compared with 50% treated with placebo in two double-blind placebo-controlled studies (EDEMA 3 and 4) (*p* = 0.03 and 0.04). The dose was 30 mg, given subcutaneously. Median (IQR) time to estimated onset of improvement, was (1.33 to >4) h with active treatment, compared with >240 (2.25 to >4) h for placebo (*p* = 0.08). Median (IQR) time to patient report that symptoms were “a lot better or resolved,” was 2.75 (1.33 to >4) h with ecallantide, compared with >4 (2.25 to >4) h for placebo (23, 24). Validated novel composite endpoints, designed to provide a holistic picture of response also favored ecallantide over placebo: treatment outcome score and mean symptom complex score both showed improvement at 4 and 24 h compared with baseline (28).

Berinert, Cinryze (except USA), Ruconest, Icatibant, and Ecallantide (USA only) are all licensed for acute treatment of hereditary angioedema (Table 1).

#### Observational Studies

Observational studies have largely confirmed results from the double-blind studies. A prospective study of Berinert (plasma-derived C1 inhibitor) 20 U/kg to treat 1,085 attacks in 57 patients over 2 years, showed onset of symptom relief after a median (range) of 0.37 (0.05–497) h and complete resolution at 14.28 h (0.17–497.0) (29). Likewise a prospective observational study of Cinryze (plasma-derived C1 inhibitor) at 1,000 U, with additional 1,000 U after 1 h if required, showed onset of relief at median 0.75 h. Time to resolution and the proportion of patients requiring the additional dose are not reported (30).

Recombinant C1 inhibitor (Ruconest) 50 U resulted in onset of symptom relief in an average 1.25 h (CI 1.32–1.48) with complete resolution at 5 h (3.52–6.1) (31, 32).

In a prospective open-label trial of ecallantide, median time to improvement was 59–113 min. 73–100% improved within 4 h, comparing favorably with the placebo-controlled trial where 69% had improved at this time point. No information concerning complete resolution is available (33).

According to the icatibant outcome survey (IOS), a disease registry for patients eligible to receive icatibant, median time to resolution was 4.4 h. This response was markedly more rapid than the median 8 h reported in double-blind studies and may reflect the ease with which icatibant, given subcutaneously, can be self-administered, thus enabling earlier treatment (34). A large Italian observational study has noted similar “real life” benefits, with time to resolution of 5 h (4).

Older retrospective case series have also provided useful information. Bork compared 17,444 untreated abdominal attacks treated with 4,834 abdominal attacks treated with C1 inhibitor concentrate in patients attending a large German center over several decades. Most patients had hospital-based treatment with Berinert (plasma-derived C1 inhibitor). Treated attacks were less severe and shorter in duration than untreated attacks. Onset of pain relief was within 2 h of C1 inhibitor in 92.6% and mean duration of attack was reduced from 92 to 39.9 h. Mean maximum pain score was reduced from 8.6 to 4.5 on a 10-point scale (9).

### Acute Attacks Occur Despite Prophylaxis

Acute attacks may occur at any site, despite use of currently available prophylaxis (35, 36).

Cinryze, 1,000 U twice weekly, has been shown in double-blind trials to reduce frequency of acute attacks by a median of 50%, also reducing severity (21). Unblinded, prospective, and retrospective observational studies, where adjustment of dose or frequency is permitted show better efficacy [(35, 37), Ref. this issue]. Nevertheless, “breakthrough” attacks occur unpredictably and may be severe. New subcutaneous prophylactic regimens, although showing a high efficacy, are still associated with occurrence of occasional attacks, which require treatment (38–40).

Oral prophylactic agents are similarly incompletely effective (Ref. this issue). Attenuated androgens such as danazol have been shown to have high efficacy at high doses. However, efficacy is reduced and varies greatly from patient to patient at the lower doses currently recommended [(41), this issue]. Antifibrinolytics, such as tranexamic acid or epsilon aminocaproic acid have unknown, but probably lower efficacy, with frequent breakthrough attacks [(42, 43), Ref. this issue].

Thus, severe attacks at any site, including life-threatening upper airway attacks, may occur despite prophylaxis. For this reason, a plan for treatment of acute attacks is strongly recommended for every patient, regardless of any prophylactic measures (11–14, 37, 44) (see Box 1).

Box 1 **Acute treatment for hereditary angioedema.**

- Every patient should have an acute care plan.
- C1 inhibitor (plasma-derived or recombinant), ecallantide, or icatibant are effective acute treatments.
- Best acute treatment should be decided on an individual basis.
- Best acute treatment may vary between attacks for a given patient.

### Optimization of the C1 Inhibitor Dose

Dose of C1 inhibitor, whether plasma-derived or recombinant, appears to be important. Blinded and unblinded trials of Ruconest (recombinant C1 inhibitor; Conestat alpha) showed a dose–response in proportion of responders at 4 h and in time to improvement and resolution, with optimum outcomes at 50–100 U/kg (45).

Double-blind trials of Berinert (plasma-derived C1 inhibitor) were consistent with a dose-dependent response for 10 and 20 U/kg compared with placebo, although only the 20 U/kg dose was significantly different from placebo (18). Owing to differences in trial methodology and endpoints, it is more difficult to compare doses of different C1 inhibitor products, and no trials offering direct comparisons have been carried out. Nevertheless, Hack et al. compared response to active C1 inhibitor treatment, adjusted for placebo response for each C1 inhibitor. Placebo response was high, with up to 50% showing improvement within 4 h, reflecting the self-limiting nature of attacks and perhaps also the subjective endpoints. Hack’s analysis suggested that percentage of subjects experiencing relief at 4 h and time to resolution were all improved with increasing dose, up to 50 U/kg, above which maximum efficacy there was no further improvement. Time to initial relief also appeared to show a dose-dependent response, at least for doses up to 25 U/kg (45) (see Box 2).

Box 2 **Dose of C1 inhibitor.**

- (There is weak evidence to suggest that) higher doses of C1 inhibitor produce more rapid response*.
- (There is weak evidence to suggest that) higher doses of C1 inhibitor are associated with higher response rates*.

*Within the licensed dose.

### Timing of Treatment

Patients experiencing HAE attacks have traditionally been asked to wait until attacks are moderate or severe, for diagnostic reasons, and in the misplaced assumption that this will minimize the use of expensive medication. More recent evidence has demonstrated that early treatment is associated with shorter time to resolution. Guidelines now recommend treatment for attacks at any site which have potential to cause pain, disfigurement, disability, or death, as early as possible (11–14, 37, 44).

A meta-analysis of two prospective trials; one double blind, one open, of Berinert (plasma-derived C1 inhibitor) 20 U/kg for 1,129 HAE attacks showed that shorter time to treatment was associated with better outcomes. Attacks treated within 6 h of onset were of significantly shorter duration than those whose treatment was delayed more than 6 h. In the double-blind study of 56 patients, earlier treatment was associated with onset of relief within a median 2.67 h (range 0.53–52) compared with 7.85 h (0.47–81.53) for those treated later, the latter being comparable with placebo in this study. Interestingly, there was no significant difference in onset of relief for delayed treatment in the unblinded trial but each hour treatment was delayed resulted in 0.2 h additional time to resolution (46).

Bork’s retrospective review of 4,834 abdominal HAE attacks treated with 500–1,000 U plasma-derived C1 inhibitor, a lower dose than currently recommended, showed that those typically treating attacks early suffered shorter duration and severity of pain compared with those treating attacks at a later stage, and a reduction in associated symptoms such as vomiting. Onset of relief in attacks treated early occurred at after a mean of 53.5 min compared with 114 min for attacks treated late (9). Kreuz et al. took this approach further, initiating one of the first C1 inhibitor self-administration programs, which enabled very early treatment. For patients with frequent attacks, an “individual replacement therapy” approach, where patients self-administered 500–1,000 U of C1 inhibitor at the first symptoms of an attack, demonstrated that attacks could effectively be aborted, although many patients on this program needed C1 inhibitor twice weekly or more frequently (47).

Trials with Cinryze (plasma-derived C1 inhibitor) showed a higher early response rate with earlier treatment. Treatment within 4 h after the onset of an HAE attack resulted in 77% of participants experiencing relief within 1 h, compared with 56% of those treated more than 4 h after attack onset (Cinryze EPAR). Interestingly in the Cinryze double-blind trial, a second 1,000 U dose of plasma-derived C1 inhibitor, given to 66% of those randomized to C1 inhibitor an hour after the first dose, resulted in a lower response rate than in the Berinert trial where participants received an initial higher dose, but in most cases a lower total dose, of 20 U/kg of plasma-derived C1 inhibitor given immediately. Although the trials are not a direct comparison, the observation is consistent with the importance of early treatment in controlling and terminating the HAE attack (18, 21).

Observational evidence of 426 icatibant-treated HAE attacks, from the IOS, demonstrated that earlier treatment with icatibant leads to faster symptom resolution and shorter overall attack duration at every time point measured. Attacks treated within an hour of onset resolved within a median of 3 h, whereas those treated later than 5 h of onset took a median time of 23.5 h (48). Similar benefits in time to resolution were noted in a later analysis of 652 patients from the same dataset (49).

In an analysis of two double-blind placebo-controlled trials of ecallantide, response rates were best when given within 2 h of symptom onset, with 71% improving within 4 h. Treatment given within 2–4 and 4–6 h of onset showed better response rates than treatment given later (50) (see Box 3).

Box 3 **Timing of treatment.**

- Earlier treatment is associated with reduction in duration of swelling.
- Earlier treatment may reduce pain and disability.
- Tests to diagnose the presence of an attack are required.

### Repeated Treatments

Hereditary angioedema is a lifelong condition and, for most, associated with multiple acute episodes. Therefore, it is important that treatments continue to be effective over the lifetime of the patient. Double-blind trials cannot feasibly address this question, which requires many years of observation. However, limited observational studies have been reassuring, showing no loss of efficacy over several treatments.

A prospective study of plasma-derived C1 inhibitor (Cinryze) showed maintained response, with no reduction in time to relief, for up to 30 attacks in 101 subjects treating a total of 609 attacks (30). Similar observations of repeated treatments with another plasma-derived C1 inhibitor (Berinert) describe good response to treatment of 1,085 attacks in 57 patients. Eighteen patients from this cohort received C1-INH concentrate for at least 15 HAE attacks over a mean duration of 34 months, without reduction in time to relief, resolution, or time between attacks (12).

A small retrospective case series of three patients describes apparent increase in C1 inhibitor requirements over a period of several years (51). However, the vast majority of retrospective observational studies of Berinert treatment, over 10 years or more, describe continued good response (9, 52–54).

A prospective observational study of recombinant C1 inhibitor (Ruconest), in 44 patients, showed no loss of efficacy over 3–5 treated attacks as measured by treatment effect questionnaire, investigator score, and patient-completed visual analog score (55). Another study showed maintained efficacy for recombinant C1 inhibitor in 62 patients with up to 8 attacks (32).

Data on repeated treatment with icatibant have been equally reassuring. Two prospective uncontrolled studies assessed response to icatibant over up to 7 years, with up to 5 and 12 treatments per patient, respectively. Response, measured by time to attack resolution and overall attack duration, did not diminish over time or with repeated treatments (56, 57).

A prospective study of ecallantide used to treat up to 13 episodes for a total of 625 episodes in 147 patients, showed maintained efficacy as measured by composite symptom scores and time to initial response (33).

### Symptom Recurrence

One potential concern with implications for quality of life, cost of treatment, and patient safety, is the requirement for more than one dose of acute treatment to resolve the attack or to treat relapse. In this respect, the C1 inhibitors appear to give the most durable response, although there is a dose-dependent effect and half-life may also be important. Thus, plasma-derived C1 inhibitor, given at 20 U/kg is associated with requirement for a second dose in only 1.1–1.9% of cases (4, 29). Recombinant C1 inhibitor, given in higher dose (4,200 U) but with a much shorter half-life is reported to have 0–6% relapse at 24 h (55). A more recent study indicated symptom recurrence at 72 h of 7.1% of those treated with the recommended dose of 50 U/kg (58). However, recombinant C1 inhibitor given at the lower dose of 2,100 U has been associated with requirement for additional dose(s) in 43% (31).

Several studies of icatibant treatment have shown requirement for a second dose of icatibant or rescue C1 inhibitor of around 10% in order to fully resolve the attack (4, 59).

2.5–10.1% of patients receiving ecallantide had likely or possible symptom relapse; 9.6–11% required a second dose at 4 h in order to resolve symptoms (60, 61).

These studies are not directly comparable but suggest general principles: individualized treatment optimization can reduce and mitigate risks of relapse. In particular, subcutaneous methods of administration and the ability to self-administer at home, reduces the impact on the patient of requiring a further treatment. Individual patient flexibility, guided by the experienced health-care professional, can enable best combination of prophylactic and acute treatments to minimize need for more than one treatment.

### Safety

Acute hereditary angioedema treatments have a good safety record. Observational trials, over many years in the case of plasma-derived C1 inhibitors, have shown good safety and tolerability (9, 29, 31–33, 52–64). Areas of concern include virus transmission for plasma-derived C1 inhibitors, antibody induction, allergic and anaphylactic reactions, and thrombosis. Each of these concerns will be addressed in the section below.

#### Virus Transmission

Modern C1 inhibitors have never been recorded as transmitting viral or prion infection in over 30 years of use. Both Berinert and Cinryze are prepared from the fractionated plasma of screened donors, and are further treated by nanofiltration and pasteurization. *In vitro* studies show effective removal of prions, and both enveloped and non-enveloped virus by these processes, providing additional evidence to back up the reassuring clinical safety record (65, 66). Nevertheless, most guidelines recommend hepatitis B vaccination and annual hepatitis B/C screening and serum save as for any patient treated with regular blood products.

#### Antibody Induction

One concern for many human replacement blood products is antibody induction.

Since patients with hereditary angioedema are heterozygotes and therefore innately tolerant to C1 inhibitor, it is unsurprising that neutralizing antibodies have not been reported, except in a unique case of a patient whose active C1 inhibitor gene contained a polymorphism (67). This is not the case with acquired C1 inhibitor deficiency where antibodies are common and in rare cases have been associated with loss of efficacy and increased C1 inhibitor requirement. These patients might be better treated with icatibant or ecallantide for acute attacks (68–70).

Non-neutralizing antibodies are relatively common, even in people who have never received exogenous C1 inhibitor. Antibody levels are reported to correlate with severity of HAE but their significance is otherwise unclear. They are not routinely measured and have no impact on efficacy or tolerability of treatment (71). Chills and hypotensive episodes have been reported when cold C1 inhibitor is given rapidly but true anaphylaxis appears vanishingly rare (52, 62).

Ruconest, a recombinant C1 inhibitor, is purified from the milk of genetically modified rabbits. Although the protein sequence is identical to that of human C1 inhibitor, post-translational glycosylation is different, leading to reductions in half-life (72). Rabbit-specific glycosylation moieties are of low-intrinsic immunogenicity and no neutralizing antibodies have been reported in patients to date (73, 74). Ruconest contains small amounts of rabbit-associated impurity. One healthy volunteer, who had undeclared severe rabbit allergy, experienced anaphylaxis on receiving Ruconest. However, no further cases of anaphylaxis or severe Ruconest allergy have been reported, including after administration to patients who retrospectively have been found to have IgE sensitization to rabbit or milk protein (45). The requirement for rabbit-specific IgE testing has recently been removed and Ruconest is now considered safe for home self-administration (75).

Ecallantide, a recombinant peptide synthesized in *Pichia pastoris* is associated with antibody induction in up to 20% of patients. Anaphylactoid reactions have been reported in 3.5% of recipients and less severe hypersensitivity is common. The etiology of these reactions is uncertain, given that they are not associated with IgE antibodies, nor with raised tryptase, as would be expected in conventional type I anaphylaxis. Subsequent tolerance is often achievable with or without desensitization. Ecallantide is currently administered by a health-care professional, usually under a home-visit scheme provided by the manufacturer (33, 76).

Icatibant is a synthetic peptide. No antibody formation or anaphylaxis has been reported to date and systemic reactions appear very rare. Local erythema and swelling at the injection site is almost universal and may relate to agonist activity due to locally high-icatibant concentration (63).

#### Thrombosis and Vascular Problems

Plasma-derived C1 inhibitors, but not recombinant C1 inhibitor, have been associated with venous and arterial thrombosis. C1 inhibitor directly regulates factors XI and XII of the coagulation system, plasminogen in the fibrinolytic system as well as exerting indirect effects *via* activities such as kallikrein inhibition. Factor XII itself has a previously under-recognized importance in generation of bradykinin (77, 78). Therefore, effects related to perturbation of coagulation and fibrinolysis would be expected. In practice, hereditary angioedema itself is not known to be associated with clinical thrombosis or bleeding tendency, despite *in vitro* abnormality. Exogenous plasma-derived C1 inhibitor has been associated with thrombosis *in vivo* at both therapeutic and supra-therapeutic doses in some but not all series (79–80). Use of in-dwelling catheters may act synergistically to increase risk of thrombosis and should be avoided (81). Difficulties with venous access are common in those severely affected but should in future be reduced by earlier use of self-administration for acute treatment and by the new subcutaneous and oral prophylactic options.

Thrombosis has not been reported with recombinant C1 inhibitor, although experience is more limited, nor with icatibant or ecallantide (63).

Observational data from the IOS has not indicated any concern regarding cardiovascular health in up to 8 years follow-up (63).

### Special Situations

#### Pregnancy and Lactation

Although there are no prospective studies of hereditary angioedema treatments in pregnancy, there is often increased requirement for acute treatments during this time (see Box 4). Pregnancy is associated with increased frequency of attacks in many but not all women, and oral prophylactic agents such as attenuated androgens or fibrinolytics are contraindicated (82, 83). Plasma-derived C1 inhibitor is identical to the patient’s endogenous C1 inhibitor and is the acute treatment of choice during pregnancy (83). Observational studies provide evidence as to its safety, particularly for Berinert (84–88).

Box 4 **Consensus: pregnancy, lactation, and childhood.**

- Plasma-derived C1 inhibitors are the treatment of choice in pregnancy.
- There are no safety concerns with recombinant C1 inhibitor in pregnancy, although experience is lacking.
- Ecallantide and icatibant should be avoided in pregnancy and during lactation because evidence of safety is lacking.
- Plasma-derived C1 inhibitors are the treatment of choice for preadolescent children.
- There are no safety concerns with recombinant C1 inhibitor in preadolescent children, although experience is lacking.
- Ecallantide and icatibant may have a role in the treatment of children. However, evidence of efficacy and safety is lacking*.

*Since the consensus meeting, evidence has been published demonstrating efficacy and safety of ecallantide and icatibant in children and adolescents.

In contrast, there is no published information about recombinant C1 inhibitor in pregnancy, beyond a single case report, although theoretically this should be safe. Icatibant and ecallantide did not show fetal toxicity in animal studies but experience is limited to case reports of icatibant use (89–91). For this reason, icatibant and ecallantide should be avoided in pregnancy and women of childbearing age counseled to this effect (83).

#### Childhood

Until recently, plasma-derived C1 inhibitors have been the treatment of choice in the under 18-age group. Few children have been included in double-blind studies and, since attacks are generally less frequent pre-puberty, very few younger children have been included. Data from unblinded and observational studies show similar responses to those of adults. Plasma-derived C1 inhibitors remain the acute treatment of choice at the present time. However, their requirement for intravenous access can be particularly problematic in children and there is considerable interest in the potential for subcutaneous options (92, 93).

46 unique subjects below the age of 18 years (2–5 years: *n* = 3; 6–11 years: *n* = 17; 12–17 years: *n* = 26) were included in the Cinryze (plasma-derived C1 inhibitor) clinical study program, mainly in the open-label studies (30). The proportion of HAE attacks achieving unequivocal relief of the defining symptom within 4 h after Cinryze treatment was comparable between the 22 children (age range 2–17 years) and 24 adults, with 89 and 86% of attacks achieving relief, respectively (30). In a smaller prospective study of nine children, 2–11 years of age, each treated for a single attack with 500–1,500 U (21–52 U/kg) of Cinryze showed a median time to relief of 0.5 h (range 0.25–2.5 h) (94).

Another plasma-derived C1 inhibitor (Berinert) was successful in reducing time to relief in seven children treated with 20 U/kg in the double-blind placebo-controlled trial. Onset of relief occurred in median 0.42 h and full resolution after 8.08 h. Similar benefit was seen for 9 children with 115 attacks treated in the open-label extension, with onset of relief in median 0.49 h and resolution after 14.1 h (95). Berinert has been used to treat children in Europe for several decades, with reassuring retrospective data reported concerning efficacy and safety (96, 97).

Ruconest appears to work well in adolescents aged 14–18 years. Analysis of its use in double-blind and open-label trials of 24 adolescents treated with 50 U/kg, 1 treated with 100 U/kg and 24 treated with 2,100 U fixed dose, shows 90% response within 4 h. Onset of relief occurred at median (CI) 2 (0.57–2.17) h, and 0.62 (0.52–1.0) h, respectively, for 2,100 U and 50 U/kg. Almost full relief occurred at 4 (4–12.2) h and 1.93 (1.35–2) h, respectively. 8% of those treated with 50 U/kg and 67% of those treated with 2,100 U received a second dose. Ruconest was well-tolerated in this group (98).

An analysis of ecallantide treatment in children aged 9–17, participating in double-blind and open-label trials of 30 mg for acute attacks, showed significantly improved response with ecallantide for 25 children experiencing 62 attacks compared with placebo (10 children and 10 attacks). Medium (IQR) time to initial improvement was 0.92 (0.53–1.95) h for ecallantide compared with 3.38 (1.4 to >4) for placebo and time to almost full resolution was 1.53 (0.52–3.5) h for ecallantide compared with 3.77 (3.3 to >4) h for placebo (99).

A small prospective study of icatibant treatment, 0.4 mg/kg, in 11 prepubertal and 11 adolescent children showed similar good response in both groups with median time to relief onset of 1.0 (95% CI 1–1.1) h and median time to minimal symptoms of 1.1 (1.2) h. All patients experienced onset of relief within 4 h. Notwithstanding the almost universal injection-site reactions, icatibant was well-tolerated (100).

## Cost of Treatment

Effective acute HAE treatments are costly to develop and to purchase, posing challenges to funding authorities. These costs have profound consequences on local funding policies which affect availability of individual drugs, often leading to barriers, delays, and inequities in access to treatment. Concerted action by patient associations, physicians, pharmaceutical companies, and governments has ensured reasonable access in high and some middle income countries, despite the extra post-licensing evidence of cost-effectiveness required by many funding bodies (5, 101).

## Discussion

Availability of evidence-based acute treatments has improved immeasurably over the past 10 years, with C1 inhibitors, icatibant and ecallantide established as practical, effective treatments. Trials directly comparing efficacy of individual agents and their suitability for particular patient groups would be desirable but are unlikely to be practical for such a rare disorder.

Access to effective treatments has enabled patients and clinicians to optimize timing of treatment and to move from a treatment model that prevents death and relieves intolerable suffering to one which aborts early symptoms, enabling fuller participation in society, and mitigating the psychological and economic burdens (3–5).

Evidence from observational data and *post hoc* analyses of double-blind trials provides additional evidence of benefits of early treatment and also the importance of adequate C1 inhibitor dose. Evidence based on such analyses is suboptimal but since further double-blind studies to answer these questions are unlikely, the HAE community must rely on the evidence that exists, which, taken together is compelling.

Many other unanswered questions exist: 7.7% of patients experience more than one attack per week (49). Little data exist on the best acute treatment for these patients. Observational studies do not show a relationship between frequency of attacks and risk of recurrence (59, 61). However, case reports suggest a high frequency of recurrence in this situation (59, 102) and it may be that an agent with a longer half-life, namely plasma-derived C1 inhibitor will continue to be the gold standard treatment for those with very frequent attacks.

Self-administration has been demonstrated to improve access to acute treatments [(15), this issue]. In order to make self-administration accessible to as many patients as possible, easier methods of administration are required and risk of recurrent symptoms need to be minimized. Subcutaneous treatments with low risk of recurrence or anaphylaxis would improve accessibility even further. Oral acute treatments remain an aspiration.

Access to effective acute treatments for HAE, suitable for administration outside the health-care environment, has revolutionized management and transformed the lives of many struggling with this debilitating and dangerous disorder. Further improvement is required. In particular, more effective prophylactic options will reduce need for emergency acute treatments.

## Author Contributions

HL performed the literature search, presented the conclusions, led the discussion with the HAWK group, and wrote the manuscript. HL acknowledges the support Professors Konrad Bork and Marc Riedl and the very helpful discussions of the HAWK group.

## Conflict of Interest Statement

HL has participated in research for, served as a consultant or speaker or received educational support from the following companies: BioCryst, CSL Behring, Pharming, and Shire (Dyax).
